# Tracking molecular resonance forms of donor–acceptor push–pull molecules by single-molecule conductance experiments

**DOI:** 10.1038/ncomms10233

**Published:** 2015-12-15

**Authors:** Henriette Lissau, Riccardo Frisenda, Stine T. Olsen, Martyn Jevric, Christian R. Parker, Anders Kadziola, Thorsten Hansen, Herre S. J. van der Zant, Mogens Brøndsted Nielsen, Kurt V. Mikkelsen

**Affiliations:** 1 Department of Chemistry, University of Copenhagen, Universitetsparken 5, Copenhagen DK-2100, Denmark; 2 Kavli Institute of Nanoscience, Delft University of Technology, GA Delft 2600, The Netherlands

## Abstract

The ability of molecules to change colour on account of changes in solvent polarity is known as solvatochromism and used spectroscopically to characterize charge-transfer transitions in donor–acceptor molecules. Here we report that donor–acceptor-substituted molecular wires also exhibit distinct properties in single-molecule electronics under the influence of a bias voltage, but in absence of solvent. Two oligo(phenyleneethynylene) wires with donor–acceptor substitution on the central ring (cruciform-like) exhibit remarkably broad conductance peaks measured by the mechanically controlled break-junction technique with gold contacts, in contrast to the sharp peak of simpler molecules. From a theoretical analysis, we explain this by different degrees of charge delocalization and hence cross-conjugation at the central ring. Thus, small variations in the local environment promote the quinoid resonance form (off), the linearly conjugated (on) or any form in between. This shows how the conductance of donor–acceptor cruciforms is tuned by small changes in the environment.

## Abstract (web summary)

Donor-acceptor molecules are important components for molecular electronics applications. Here, the authors show that donor-acceptor molecular wires exhibit changes in conductivity under the influence of small changes in the environment.

## Graphical abstract

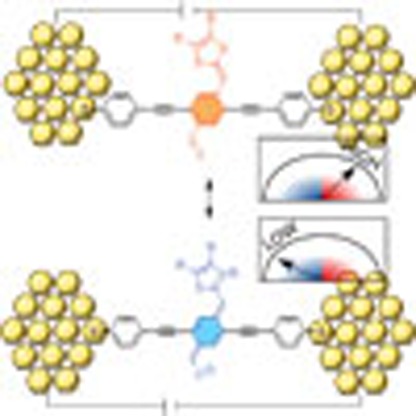

π-Conjugated molecules such as oligo(phenyleneethynylene)s (OPEs) have been explored extensively as molecular wires in molecular electronics^1^,^2^,^3^,^4^,^5^,^6^. The single-molecule conductance depends on the nature of the π-system, and, in particular, both theoretical^7^,^8^ and experimental^9^,^10^,^11^,^12^ studies have revealed that cross-conjugated molecules transmit current much less efficiently than linearly conjugated ones. Molecules for which the conjugation pathway along the molecular wire can be changed by external stimuli thus present convenient on/off switches for molecular electronics devices^12^. Another important design element in molecular electronics is the ability to generate unimolecular rectifiers by linking together an electron donor (D) and acceptor (A) as proposed theoretically by Aviram and Ratner^13^ in 1974 and later verified experimentally for various systems^4^,^14^,^15^,^16^,^17^. While the Aviram–Ratner diode has the donor (tetrathiafulvalene) and acceptor (tetracyanoquinodimethane) units as parts of the actual wire, we became interested in placing D–A units orthogonally to an OPE3 molecular wire, incorporating thioacetate end groups to allow anchoring to gold electrodes. The rationale behind this design is to promote quinoid character, that is, cross-conjugation, along the molecule as a function of the degree of D–A charge delocalization in the ground state.

The unsymmetrical cruciform-like OPE3 molecules **1** and **2** shown in Fig. 1 present examples of push–pull D-A molecules. They incorporate the dithiafulvene unit (DTF ∼ ‘tetrathiafulvalene half unit') as electron donor and either an aldehyde or indan-1,3-dione as electron acceptor. For these two molecules, a quinoid and zwitterionic resonance form can be drawn, corresponding to a cross-conjugated pathway along the OPE wire as schematically shown in Fig. 1 for molecule **2**, and promoted by the formation of an aromatic 1,3-dithiolium ring^18^. The contribution of such a cross-conjugated resonance form to the overall resonance hybrid is expected to reduce the conductance along the wire. In contrast, we have recently shown that placing one DTF unit along the OPE3 (molecule **3**) does not significantly alter the single-molecule conductance relative to that of the **OPE3** when recorded by scanning tunneling microscopy (STM) or mechanically controlled break-junction techniques^19^. The question we therefore set out to answer is the following: How sensitive is the conductance of a D–A molecule to the local environment, which may promote electron delocalization of the molecule towards one or the other of the two resonance forms shown in Fig. 1? As the environment of a molecule in a break-junction at a certain bias may change from one opening–closure experiment to the other and from one electrodes position to the other along the same opening experiment, this technique was chosen to track variations in conductance. We here present the synthesis followed by experimental and theoretical electron transport studies of molecules **1** and **2**. These D–A cruciform molecules are found to exhibit remarkably broad conductance histograms. The molecular wire **4** represents a borderline case for cross-conjugation used in the theoretical study, which also covers results on the protonated form **1**·H^+^.

## Results

### Synthesis

The synthesis of molecules **1** and **2** is shown in Fig. 2. First the known dialdehyde **5** (ref. 20) was treated with one equivalent of the phosphonium salt **6** (ref. 21) and triethylamine to furnish the product **7** where one DTF unit has been introduced. Subjecting this compound to potassium carbonate and 1-propanol resulted in both desilylation and transesterification of the peripheral methyl ester substituents to propyl esters, providing better solubility. The resulting product **8** was then subjected to Sonogashira cross-coupling reactions with *S*-(4-iodophenyl) ethanethioate^22^, furnishing the OPE3 **1**. Finally, a Knoevenagel condensation between **1** and indan-1,3-dione gave the OPE3 **2**.

### Donor–acceptor properties in bulk media

The structure of **7** was confirmed by X-ray crystallographic analysis (Fig. 3). The molecule exhibits quinoid character characterized by values of 0.03 and 0.02 Å for the two asymmetric parts (calculated as (*a*+*b*−2*c*)/2, where *a*, *b* and *c* are the lengths of the three C−C bonds in the ring between the DTF and CHO units; due to asymmetry of the molecule, two values are calculated). This corresponds to roughly 15% of the quinoid character of *p*-benzoquinone, which has a quinoid character of 0.16 Å (ref. 23). Molecules **1** and **2** exhibit longest wavelength absorption maxima in CH_2_Cl_2_ at ca. 422 nm (*ɛ*=2.4 × 10^4^ M^−1^ cm^−1^) and 501 nm (*ɛ*=3.0 × 10^4^ M^−1^ cm^−1^), respectively (Fig. 4). These absorptions, which are rather broad, show weak solvatochromic behaviour and are assigned as charge-transfer absorptions. They are thus blueshifted to ca. 416 nm (**1**) and 480 nm (**2**) in the more polar solvent MeCN. These blueshifts indicate some charge delocalization in the ground state, which is hence stabilized slightly more than the excited state by a polar solvent. Molecule **2** exhibits the lowest energy of the charge-transfer transition, which signals both the stronger acceptor strength of the indan-1,3-dione unit and a weaker electronic coupling in the ground state between donor and acceptor units^24^—as reflected by a smaller ground-state dipole moment of **2** than of **1** (3.04 D versus 5.48 D, see calculations below). Charge delocalization in the ground state of **1** was further corroborated from electrochemistry. Thus, compound **7** (representing the central part of **1**) showed an irreversible oxidation at *E*_pa_=0.88 V versus Fc/Fc^+^ in CH_2_Cl_2_ (0.1 M Bu_4_NPF_6_) according to cyclic voltammetry (CV) and at ca. 0.82 V versus Fc/Fc^+^ according to differential pulse voltammetry (DPV); see Supplementary Figs 1 and 2. For comparison, under the same conditions a related compound with one DTF unit, but no aldehyde acceptor unit, was oxidized irreversibly at +0.75 V (CV)/+0.70 V (DPV)^19^. Despite the irreversible character (explained by radical dimerization^25^,^26^,^27^), it is clear that the electron-withdrawing aldehyde functionality has decreased the donor strength of the DTF unit noticeably (>100 mV), signalling an important contribution of the quinoid resonance form to the overall resonance hybrid. Compound **7** experienced an irreversible reduction at *E*_pa_=−1.84 V (CV)/−1.69 V (DPV) versus Fc/Fc^+^ (Supplementary Figs 3 and 4).

### Single-molecule conductance measurements

D–A molecules **1** and **2**, together with the reference molecules **3** and **OPE3** (previously studied^19^), were subjected to single-molecule conductance measurements employing the mechanically controlled break-junction (MCBJ) technique with gold contacts. Briefly, a MCBJ sample consists of a gold wire lithographically defined on a flexible and electrically insulated phosphorous bronze substrate. By bending the device in a three-point bending configuration, the wire breaks at the thinnest part defined in the middle and the two broken extremities can be used as tips to contact single molecules. After the characterization of the gold device, we deposit the molecules of interest by drop casting a 2 μl droplet of a 1 mM solution in CH_2_Cl_2_ onto the MCBJ sample. We then perform conductance measurements (after the solvent has evaporated) as a function of electrode displacement and in the following we will illustrate the measurement in the case of molecule **2**. Figure 5a presents a typical conductance trace recorded in presence of molecule **2** while stretching the nanoelectrodes. The bias voltage is 0.1 V and the electrodes speed is 5 nm s^−1^. All the traces show a sharp drop in conductance just after 1 G_0_ attributed to the rupture of the gold wire and the rearrangement of the apex atoms. The presence of plateaus in conductance ∼10^−4^ G_0_ indicates the formation of molecular junctions that can be stretched for a distance larger than 1 nm (three leftmost traces in Fig. 5a). On the contrary, empty junctions, where no molecules are trapped, show below 1 G_0_ an exponential decay of the conductance as a function of the distance (the two traces on the right of Fig. 5a). We notice that some of the conductance plateaus present large fluctuations around the value of 10^−4^ G_0_. We measure >1,000 consecutive of such conductance versus stretching traces and we compile conductance histograms.

Figure 5b shows the conductance histograms of **1**, **2** and **OPE3** built by logarithmically binning all the individual conductance traces. In each of the three histograms, a peak—representing the most probable conductance values—is visible starting at the same high-conductance value ∼10^−3^ G_0_. From an inspection of the different distributions we notice that the peak for the D–A molecules **1** and **2** is much wider than the peaks of **OPE3**. The conductance histogram of molecule **3**, plotted in Fig. 5c, presents a peak similar to **OPE3**. When analysing the different molecular conductance peaks, we find that only the conductance peak of **OPE3** can be fitted to a single Gaussian, while, to achieve a good fit of the conductance distributions of the D–A molecules **1** and **2**, a sum of at least two Gaussian peaks is necessary defined as:


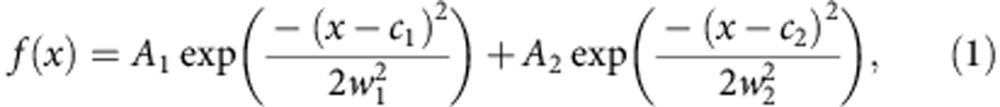


where *c*_1/2_ and *w*_1/2_ represent the centre and the s.d. (corresponding to the half-width at half-maximum divided by 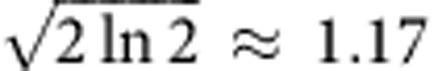) of the Gaussian peaks. From the fit parameters reported in Table 1, we see that all the three distributions present a Gaussian peak centred between 1.35 × 10^−4^ and 1.89 × 10^−4^ G_0_ and with a s.d. of 0.45 decade in the logarithmic conductance. Thus, this ‘high' conductance peak corresponds to a configuration that is present both in **OPE3** and in the D–A molecules **1** and **2**. In the case of the D–A molecules we find a second peak located around 10^−5^ G_0_, about one order of magnitude lower in conductance in respect to the first peak. This peak is considerably wider than the ‘high' conductance peak and is absent in the **OPE3** conductance distribution. For further details on the measurements, see Supplementary Figs 5–7, and for further details on the fits, see Supplementary Fig. 8 and Supplementary Methods.

As mentioned earlier, the distributions of the high-conductance values are approximately the same in the four OPE3 molecules. To quantify the peak width, we fit each individual molecular peak to a two-piece normal distribution^28^, consisting of the halves of two normal distributions:


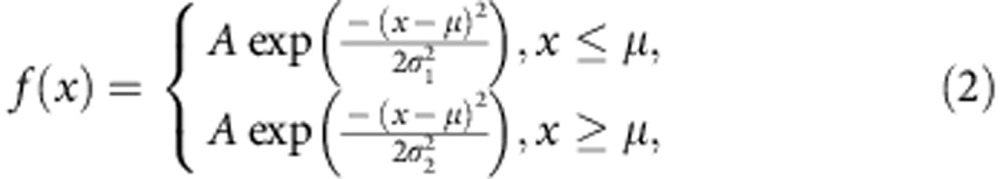


where *μ* is the mode of the distribution and *σ*_1_ (*σ*_2_) is the s.d. of the low-conductance (high conductance) part. For a symmetric distribution, the two widths are the same. From the fit we find that the maximum of the distribution is similar for all four molecules ranging from 0.9 × 10^−4^ G_0_ to 1.6 × 10^−4^ G_0_ (Supplementary Fig. 6). The widths of the conductance distributions, shown in Fig. 5d, reveal an interesting trend. When going from **OPE3** to **3**, **2** and **1**, the molecular peak gets wider in an asymmetric way. While the high-conductance width remains almost constant, the low-conductance part shows a large increase going from 0.5 decades in the case of **OPE3** to >1.5 decades for the two D–A molecules **1** and **2**. The results from the analysis of the molecular conductance distributions with the two convoluted Gaussian peaks and with the two-piece Gaussian distribution strongly suggest the presence of two states of the molecule, each with a particular most probable conductance value. For molecule **1** and **2**, the difference in conductance value is about an order of magnitude.

Two-dimensional histograms support these observations. In Fig. 6 we plot the histograms for molecules **1** and **2** where the colours represent the number of counts. In both cases the molecule appears as a broad high-counts (red) region ∼10^−4^ G_0_ that extends for >1 nm. The high-conductance values explored by the molecular junctions are comparable to the conductance of the non D–A active molecules **OPE3** and **3** (ref. 19). The length of this region is also similar and is related to the length of the molecules (∼2 nm from sulfur to sulfur). The conductance in Fig. 6 shows a broad distribution and counts below 10^−4^ G_0_. These lower conductance values (yellow/red spots) appear below the flat region, not present for **OPE3**. Central D–A cores thus promote fluctuations to lower conductance values than for simple OPE3 molecules.

To elucidate the dependence of the electric field, D–A molecule **2** was next studied at a higher bias potential, 0.45 V. Figure 7 shows the conductance histogram at this bias potential in comparison to one at 0.1 V. It should be noted that the histograms come from a different sample than that corresponding to Figs 5 and 6, and, as the junction formation probability was lower, only by selecting the 8% traces with plateaus, we arrive at the histogram in Fig. 7. Nevertheless, at the high bias potential, the low-conductance peak is more prominent, which we take as further support for promotion of the cross-conjugated resonance form. The individual traces (Supplementary Fig. 7) show strong fluctuations in conductance (up to few orders of magnitude). In addition, the junction formation probability increased from 8% at bias potential of 0.1 V to 43% at bias potential of 0.45 V. This observation may again be related to the dipole moment of the molecule.

### Theoretical modelling

**Figure 1:**
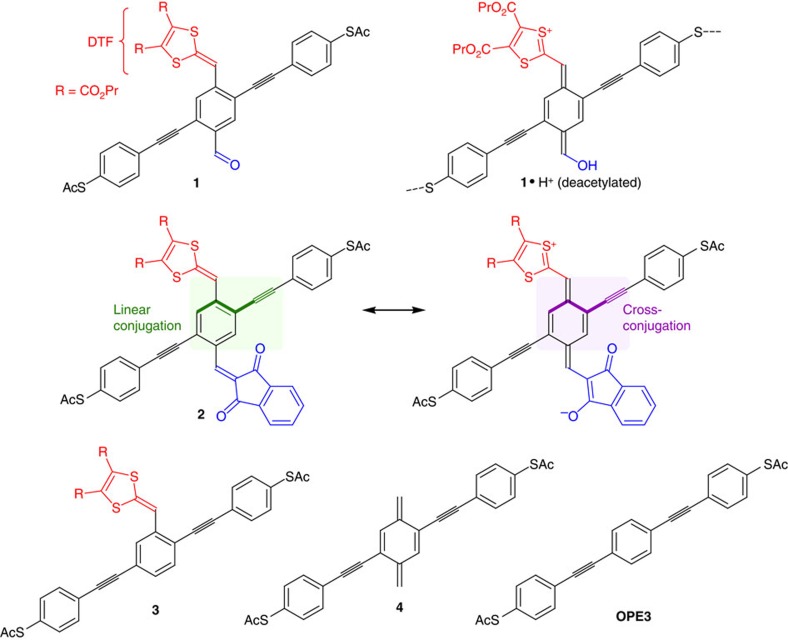
Structures of donor (red)–acceptor (blue) functionalized molecular wires. The molecules are based on a central oligo(phenyleneethynylene) (OPE3) core with acetyl (Ac) protected thiolate end groups for electrode anchoring. Dithiafulvene (DTF) is used as the donor unit. Protonation of **1** should promote the cross-conjugated and quinoid resonance form (**1**·H^+^). Linearly and cross-conjugated resonance forms are shown for molecule **2**. Pr, propyl.

**Figure 2:**
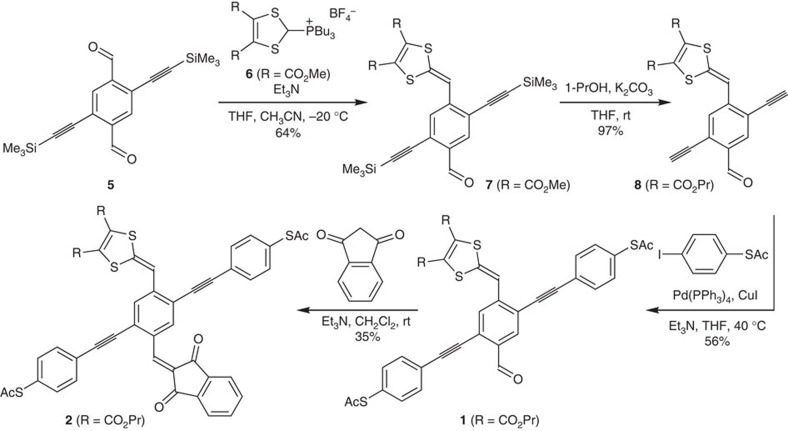
Synthesis of donor–acceptor cruciform-like molecules. The initial step is a Wittig reaction, which is followed by a one-pot transesterification and desilylation. The terminal alkyne product is subjected to a Sonogashira reaction, furnishing the molecular wire with thioacetate end groups. The last step is a Knoevenagel condensation. Me, methyl; Et, ethyl; Pr, propyl; Bu, butyl; Ac, acetyl; Ph, phenyl; THF, tetrahydrofuran; rt, room temperature.

**Figure 3:**
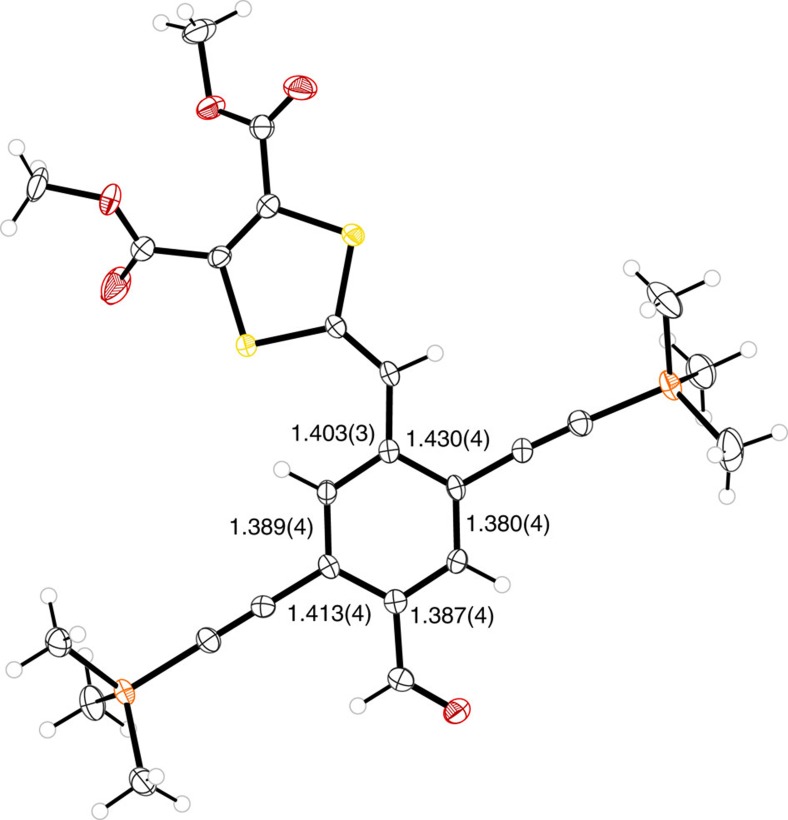
Molecular structure of 7 from X-ray crystallographic analysis. The structure includes carbon-carbon bond lengths of the central benzene ring (Supplementary Data 1). Crystals were grown from CH_2_Cl_2_/heptane. CCDC 1023660 contains the supplementary crystallographic data.

**Figure 4:**
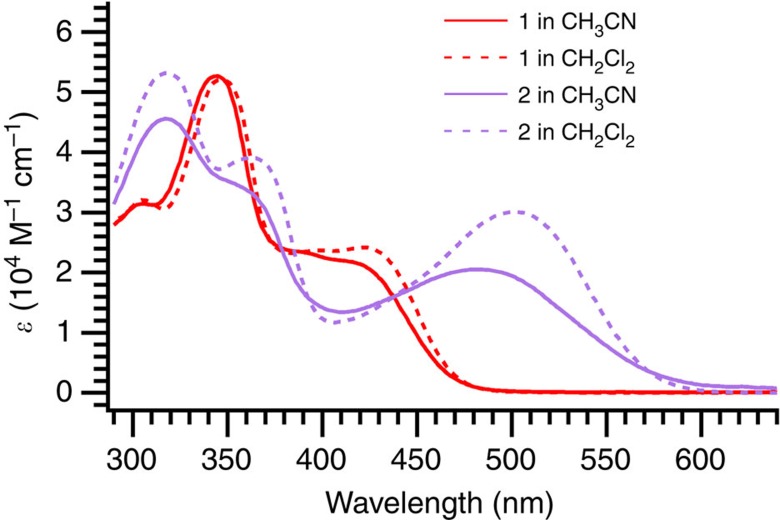
Ultraviolet–visible absorption spectra of D–A molecules 1 and 2. Spectra in two different solvents (acetonitrile and dichloromethane) are shown.

**Figure 5:**
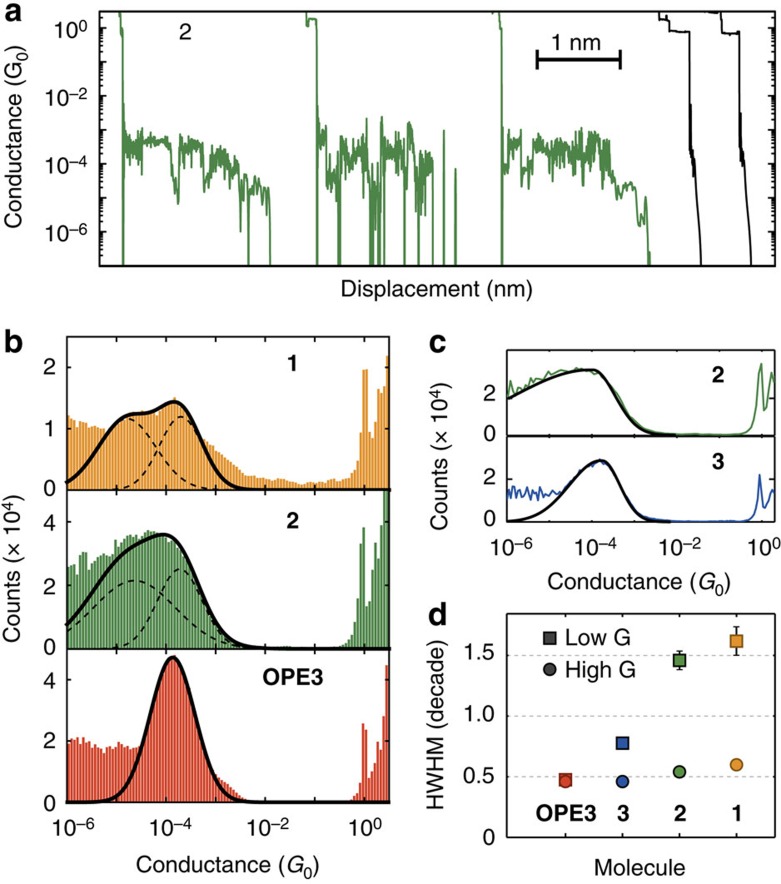
Single-molecule conductance results. (**a**) Conductance traces measured in presence of **2** while separating the mechanically controlled break-junction (MCBJ) nanoelectrodes. The bias is 0.1 V and the electrodes are retracted at a speed of 5 nm s^−1^. (**b**) Conductance histograms of **1**, **2** and **OPE3** built from >1,000 traces each. The thick black lines represent the fit of the molecular peak to a sum of two Gaussian curves defined in equation (1) and the dashed lines represent the individual Gaussian peaks. (**c**) Conductance histograms of **2** and **3** built from >1,000 traces each. The black lines represent a fit to the two-piece Gaussian function defined in equation (2). (**d**) Half-width at half-maximum of the molecular conductance peaks on the higher conductance side of the peak (circles) and lower (squares) extracted from the two-piece Gaussian standard deviations. While the high-conductance values are close to 0.5 decades for all four molecules, the donor–acceptor molecules have a much larger width at the low-conductance side.

To seek an understanding of the origin of the fluctuations in conductance values for the D–A substituted OPE3s, density functional theory (DFT) calculations were performed. The chemical view of the resonance hybrid can from a theoretical point of view be considered as a superposition of two configurations, Ψ=*C*_A_|A>+*C*_B_|B> , where the coefficients *C*_A_ and *C*_B_ give the relative distribution between the two resonance forms A and B. The two contributing states should be modelled with the exact same geometry but with varying electron distributions. ‘Linearly' conjugated structures were obtained from a potential energy surface search (and hence not solely linearly conjugated, but allowed quinoid character in the optimization), whereas ‘cross-conjugated' structures were generated from the linearly conjugated structures with an applied field from a dielectric medium (CH_2_Cl_2_, dielectric constant of 8.93 at 25 °C). Thus, the cross-conjugated and linearly conjugated molecular structures are identical in regard to geometries but have different charge distributions. Charge delocalization is promoted in the cross-conjugated structures as schematically illustrated in Fig. 8 and evident when comparing the dipole moments listed in Table 2 for the two structures of **1** and **2**. It is important to note that these structures (‘linear' and ‘cross') do not represent the fully linearly and cross-conjugated resonance forms; that is, they are not identical to the two border-line electron distributions (as drawn for **2** in Fig. 1). While the dipole moment of **2** in its ‘linearly' conjugated ground state (3.04 D) is not significantly different from that of **3** (3.61 D), the dipole moment of **1** is significantly larger (5.48 D), and molecule **1** should indeed be described by a hybrid of linearly and cross-conjugated resonance forms in the ground state (in accordance to the results from studies in bulk media). For selected Mulliken charges of **1** and **2**, see Supplementary Table 1.

The appearance of a broad conductivity peak based on various degrees of charge delocalization within the D–A unit is supported by transmission calculations made in ARTAIOS (using a junction of two gold clusters, each of nine atoms), where the relative transmission (*T*_rel_, Table 2) is expressed as the transmission of the given structure compared with the borderline resonance form given by the cross-conjugated OPE3 **4**. It is observed that all ‘linearly conjugated' structures have similar transmissions as the parent OPE3 system (**OPE3**), which also explains the same off-set in the conductance histogram. Instead, the transmission of the cross-conjugated molecule **4** is a factor of ∼4 lower than the linearly conjugated systems. We also performed transmission calculations on the protonated form of **1** (**1**·H^+^) as protonation of the aldehyde oxygen atom should promote the cross-conjugated resonance form with a positively charged, aromatic 1,3-dithiolium ring (Fig. 1). Indeed, we find a transmission for this species, which is close to that of the inherently cross-conjugated molecule **4** (Table 2).

## Discussion

**Figure 6:**
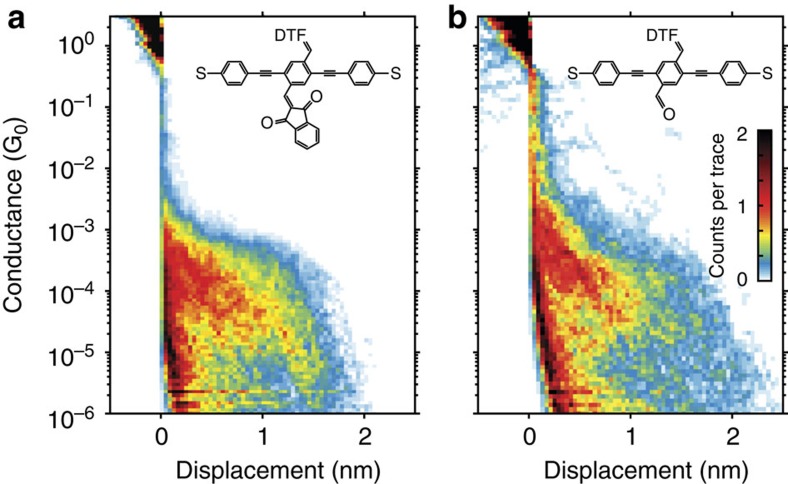
Two-dimensional conductance histograms. (**a**) Histogram of **2**; (**b**) histogram of **1**. The conductance is logarithmically binned with 16 bins per decade while the displacement is linearly binned with 31 bins per nm.

**Figure 7:**
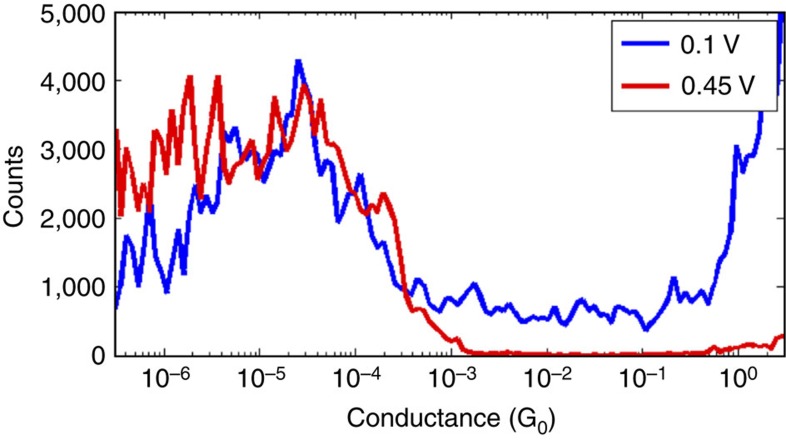
Conductance measurements at two different bias potentials. Conductance histograms of molecule **2** built from breaking traces, showing a molecular plateau, recorded at a bias of 0.1 and 0.45 V.

**Figure 8:**
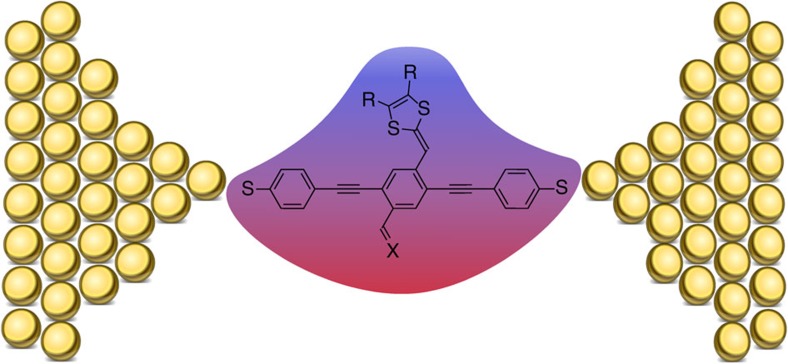
Schematic illustration of charge delocalization in donor–acceptor cruciform molecule. Small local variations due to a electric field from the gold electrodes could promote electron delocalization of the molecule towards the cross-conjugated form. Thus the charge distribution would move from the donor towards the acceptor (CH=X) end. Blue colour represents a positively charged region, while red colour represents a negatively charged region of the push–pull donor–acceptor molecule.

While the calculations support that the linear and cross-conjugated forms, promoted by various local environments, could be the origin of the broad conductance histograms of the D–A molecules, we will now discuss some other possible explanations. Thus, crosstalk and cooperative effects^29^,^30^,^31^,^32^ for transport mechanisms between molecular wires connected to the same gold electrodes is relevant to consider. A theoretical study by Dubi^32^ is able to explain a lower conductance of molecules in self-assembled monolayers^33^ by in-plane dephasing, while Akkerman and de Boer^34^ first of all ascribe differences between single-molecule and self-assembled monolayer experiments to a difference in contacts. First, cooperativity effects could result from contacting of an aggregate of molecules in the junction. Yet, the histogram lineshapes of the cruciform donor–acceptor molecules closely follow the **OPE3** line shape on the right (high-conductance part), suggesting structural similarity of junctions, supporting that the measurements on the D–A molecules are indeed on single molecules. Simulated lineshapes^30^,^31^ for molecules exhibiting cooperative effects do not agree with the measured lineshapes. In addition, the experiment showed a maximum length of the traces in the case of **2** between 1.5 and 1.8 nm for every conductance (Fig. 6). The simple **OPE3** shows a very similar situation with traces up to 1.5–2 nm. Yet, although only a single molecule seems to be contacted by the two electrodes (hence excluding in-plane dephasing effects), physisorbed molecules in the vicinity (may be assembled by π−π stacking) or attached to only one of the electrodes could change the dielectric constant of the environment influencing the molecule. The fluctuations observed for D–A molecules **1** and **2** could then be caused by interaction with aggregates of lower order for these molecules due to the bulky DTF-CO_2_Pr units, while **OPE3** may form more ordered aggregates. This effect basically contributes to the overall variation in the local environment and is thus in line with promotion of either linear- or cross-conjugated resonance forms of the contacted molecule. It must, however, be rather small as molecule **3** behaved very similarly to the simple **OPE3** although it has a central DTF-CO_2_Pr unit and only differs from molecule **1** by not having the CHO substituent. When it comes to molecule–electrode contacts, we have only changed the central part of the molecules relative to **OPE3**, but not the anchoring groups, and therefore it is unlikely that the broad conductance distributions of **1** and **2**, not experienced by **3**, originate from different molecule–electrode contacts. The central part could play a role still for contacting, but again, molecules **1** and **3** only differ by the small CHO substituent present in **1**, but have very different conductance histograms.

Another explanation for the broad conductance peaks could be the presence of different conformers, interconverted by rotation of the individual phenylene units. Seldenthuis *et al.*^35^ have previously proposed an all-electric single-molecule motor based on rotation of a permanent dipole in a modulated electric field. An oscillating electric field would cause rotation of the central dipole moiety and, in consequence, the conductance would oscillate, reaching a minimum when the central dipole moiety becomes orthogonally oriented relative to the conjugated wire. Variations in the local environment could possibly promote one conformer over the other of molecules **1** and **2**, with dipole moments of 5.48 and 3.04 D, respectively. Yet, as molecule **3** has a similar dipole moment (3.61 D) as that of **2**, but does not show the same broad conductance, an explanation based on a freely rotating, central dipole moiety seems unlikely.

In conclusion, the remarkably broad conductance histograms measured for two donor–acceptor-functionalized OPE3 molecular wires, with the donor unit being a dithiafulvene, seems to be best explained by local variations in the electric field experienced by the molecules, which promotes one resonance form over the other as the most significant contributor to the resonance hybrid. This explanation does not exclude that other effects could be active as well. The upper limit to the conductance should thus correspond to a linearly conjugated resonance form similar to a simple, unsubstituted OPE3, while the lower limit should correspond to a fully cross-conjugated resonance form. In other words, the central donor–acceptor-substituted benzene ring acts as a relay for on/off conductance. This is to our knowledge the first time, molecular resonance forms have basically been tracked by single-molecule conductance measurements. In future work we hope to be able to gain control over the conductance switching. One way of doing so may be to incorporate the D–A molecules in a three-terminal device (with source, drain and gate electrodes) and then subjecting the molecules to various gate potentials at low temperature.

## Methods

### Theory

Geometry optimizations were performed in Gaussian09 (ref. 36) with DFT/CAM-B3LYP/cc-pVDZ. For the charge-separated structures, the Polarizable Continuum Model was used with a dielectric constant of 8.93 at 25 °C with cavity size corresponding to van der Waal's radii^37^,^38^. All transmission calculations have been performed with ARTAIOS^39^ based on a DFT single-point energy calculation from Gaussian09 with CAM-B3LYP/Def2TZVP on the entire junction consisting of the molecule and two gold clusters each of nine gold atoms^40^. The Def2TZVP basis set uses effective core potentials on the gold atoms. The electronic structure calculation is combined with a wideband approximation spectral density, which means that for the transport calculation the number of gold atoms is not important.

### Break-junction experiments

Single-molecule electrical transport measurements were performed with mechanically controlled break junctions employed with gold electrodes. The low-noise electronics used to apply the bias voltage and measure the current is home-made and we control it through Python and Adwin routines. We use a sampling rate of 500 Hz and each conductance point is averaged over 256 samples such that the resolution of the analogue-to-digital conversion increases from 16 bits to 20 effective bits.

### Synthesis

General methods: all reactions were performed under inert atmosphere under an Ar atmosphere, whilst the purification of the reaction mixtures was performed in ambient air. In the case of palladium-catalyzed reactions, all solvents were degassed with Ar for 20 min in an ultrasonic bath. Chemicals were used as purchased from commercial sources. Tetrahydrofuran (THF) was distilled from sodium/benzophenone couple. Flash column chromatography was performed using silica gel (40–63 μm). Thin-layer chromatography (TLC) was carried out using aluminium sheets precoated with silica gel (silica gel 60 F_254_). ^1^H NMR (500 MHz) and ^13^C NMR (125 MHz) spectra were recorded on an instrument with cryoprobe using the residual solvent as the internal standard (CDCl_3_, ^1^H 7.26 p.p.m. and ^13^C 77.16 p.p.m.; CD_2_Cl_2_, ^1^H 5.32 p.p.m. and ^13^C 54.00 p.p.m.). All chemical shifts are quoted on the *δ* scale (p.p.m.), and all coupling constants (*J*) are expressed in Hz. For NMR spectra, see Supplementary Figs 9 and 10 (compound **1**), Supplementary Figs 11 and 12 (compound **2**), Supplementary Figs 13 and 14 (compound **7**) and Supplementary Figs 15 and 16 (compound **8**). High-resolution mass spectra were recorded in positive mode on a MALDI FT-ICR instrument equipped with a 7 T magnet, using dithranol as the matrix. Melting points are uncorrected. Ultraviolet–visible spectroscopic measurements were performed in a 1-cm path length quartz cuvette and all ultraviolet–visible spectra are solvent corrected. Elemental analysis was performed at London Metropolitan University.

### Dimethyl-2-{4-formyl-2,5-bis[(trimethylsilyl)ethynyl]benzylidene}-1,3-dithiole-4,5-dicarboxylate (7)

To a solution of **5** (1.20 g, 3.68 mmol) and **6** (2.08 g, 4.21 mmol) in a dry THF (90 ml) and CH_3_CN (30 ml) at −20 °C was added slowly Et_3_N (5 ml). The cooling bath was removed and stirring continued for 45 min. The reaction was quenched by the addition of H_2_O (200 ml) and was diluted with CH_2_Cl_2_ (500 ml). The organic phase was separated and dried over Na_2_SO_4_, filtered and concentrated *in vacuo*. Purification by flash column chromatography (SiO_2_, gradient elution 40–100% CH_2_Cl_2_/heptane) gave **7** as a bright orange solid (74 mg, 64%). Melting point: 194 °C (decomp); TLC (CH_2_Cl_2_:heptane 50:50 v/v): *R*_f_=0.20; ^1^H NMR (500 MHz, CDCl_3_): *δ* 10.41 (s, 1H), 7.98 (s, 1H), 7.44 (s, 1H), 6.95 (s, 1H), 3.89 (s, 3H), 3.88 (s, 3H), 0.30 (s, 9H), 0.27 (s, 9H); ^13^C APT NMR (125 MHz, CDCl_3_): *δ* 190.32, 159.82, 159.76, 141.69, 138.09, 133.08, 131.73, 131.65, 130.16, 128.78, 126.52, 121.85, 111.75, 104.43, 103.60, 101.78, 99.87, 53.72, 53.68, −0.05, −0.10; HRMS (*m/z*): 551.08089 [M+Na]^+^; calcd. for C_25_H_28_O_5_S_2_Si_2_Na: 551.08137; elemental analysis (%): C 56.67, H 5.42; calcd. for C_25_H_28_O_5_S_2_Si_2_: C 56.78, H 5.34. X-ray quality crystals were grown from a CH_2_Cl_2_/heptane solution.

### Dipropyl-2-(2,5-diethynyl-4-formylbenzylidene)-1,3-dithiole-4,5-dicarboxylate (8)

A mixture of **7** (282 mg, 533 μmol) and K_2_CO_3_ (469 mg, 3.36 mmol) in dry THF (10 ml) and 1-propanol (15 ml) was stirred for 3 h at room temperature. The reaction mixture was diluted with CH_2_Cl_2_ (50 ml) and filtered through a short silica plug, washing with CH_2_Cl_2_. The solvent was removed *in vacuo* giving **8** as an orange solid (228 mg, 97%). Melting point: 103–104 °C (decomp); TLC (CH_2_Cl_2_:heptane, 70:30 v/v): *R*_f_=0.40; ^1^H NMR (500 MHz, CDCl_3_): *δ* 10.40 (s, 1H), 8.04 (s, 1H), 7.56 (s, 1H), 6.97 (s, 1H), 4.24 (t, *J*=6.7 Hz, 2H), 4.23 (t, *J*=6.7 Hz, 2H), 3.55 (s, 1H), 3.48 (s, 1H), 1.70–1.78 (m, 4H), 0.99 (t, *J*=7.4 Hz, 6H); ^13^C APT NMR (125 MHz, CDCl_3_): *δ* 189.74, 159.41, 159.28, 142.12, 139.39, 133.23, 132.50, 131.91, 130.09, 129.48, 125.70, 121.16, 110.79, 85.80, 85.35, 80.52, 79.08, 68.71, 68.67, 21.93, 21.91, 10.46, 10.43; *λ*_max_ (MeCN): 407.5 nm (*ɛ*=1.89 × 10^4^ M^−1^ cm^−1^); *λ*_max_ (CH_2_Cl_2_): 415 nm (*ɛ*=2.45 × 10^4^ M^−1^ cm^−1^); *λ*_max_ (PhMe): 415 nm (*ɛ*=2.37 × 10^4^ M^−1^ cm^−1^); *λ*_max_ (cyclohexane): 397 nm (*ɛ*=2.50 × 10^4^ M^−1^ cm^−1^), 416.5 nm (*ɛ*=3.01 × 10^4^ M^−1^ cm^−1^); HRMS (*m/z*): 463.06443 [M+Na]^+^; calcd. for C_23_H_20_O_5_S_2_Na: 463.06543; elemental analysis (%): C 62.62, H 4.68; calcd. for C_23_H_20_O_5_S_2_: C 62.71, H 4.58.

### Dipropyl-2-(2,5-bis{[4-(acetylthio)phenyl]ethynyl}-4-formylbenzylidene)-1,3-dithiole-4,5-dicarboxylate (1)

To a deaerated mixture of **8** (181 mg, 411 μmol), *S*-(4-iodophenyl) ethanethioate (344 mg, 1.24 mmol), Pd(PPh_3_)_4_ (47.7 mg, 41.3 μmol), and CuI (8.80 mg, 46.2 μmol) was added a deaerated solution of Et_3_N (1 ml) in dry THF (100 ml). The resulting mixture was stirred for 18 h at 40 °C, after which time TLC analysis indicated an incomplete reaction. To the reaction flask was added Et_3_N (0.3 ml), Pd(PPh_3_)_4_ (49.5 mg, 42.8 μmol), and *S*-(4-iodophenyl) ethanethioate (114 mg, 411 μmol) and stirring was resumed for a further 3 h at 40 °C. After this period, another portion of *S*-(4-iodophenyl) ethanethioate (199 mg, 428 μmol) was added to the vessel and the reaction mixture was stirred 15 h at 40 °C. The mixture (at ambient temperature) was filtered through a short Celite plug and the solvent was removed under reduced pressure. The crude residue was purified by flash column chromatography (SiO_2_, 4% EtOAc/toluene) to afford **1** as an orange solid (170 mg, 56%). Melting point: 143–145 °C (decomp); TLC (EtOAc:toluene, 4:96 v/v): *R*_f_=0.31; ^1^H NMR (500 MHz, CD_2_Cl_2_): *δ* 10.51 (s, 1H), 8.09 (s, 1H), 7.68–7.58 (m, 5H), 7.46 (m, 4H), 7.09 (s, 1H), 4.22 (t, *J*=6.7 Hz, 4H), 2.44 (2 × s, 6H), 1.73 (tq, *J*=6.7, 7.4 Hz, 4H), 0.98 (t, *J*=7.4 Hz, 6H); ^13^C APT NMR (125 MHz, CD_2_Cl_2_): *δ* 193.65, 193.55, 190.12, 159.83, 159.65, 142.03, 139.67, 134.99, 133.30, 132.81, 132.66, 132.39, 132.29, 130.47, 130.30, 129.91, 129.38, 126.54, 124.08, 123.75, 122.00, 111.43, 97.23, 96.93, 88.50, 86.99, 69.13, 69.04, 30.74, 30.73, 22.35, 22.32, 10.65 (2 C masked); *λ*_max_ (MeCN): 344 (*ɛ*=5.27 × 10^4^ M^−1^ cm^−1^), 390 (*ɛ*=2.34 × 10^4^ M^−1^ cm^−1^), 416 nm (*ɛ*=2.18 × 10^4^ M^−1^ cm^−1^); *λ*_max_ (CH_2_Cl_2_): 347 (*ɛ*=5.21 × 10^4^ M^−1^ cm^−1^), 398 (*ɛ*=2.37 × 10^4^ M^−1^ cm^−1^), 422 nm (*ɛ*=2.41 × 10^4^ M^−1^ cm^−1^); *λ*_max_ (PhMe): 349 (*ɛ*=5.15 × 10^4^ M^−1^ cm^−1^), 397 (*ɛ*=2.37 × 10^4^ M^−1^ cm^−1^), 423 nm (*ɛ*=2.33 × 10^4^ M^−1^ cm^−1^); HRMS (*m/z*): 763.09231 [M+Na]^+^; calcd. for C_39_H_32_O_7_S_4_Na: 763.09437; elemental analysis (%): C 63.16, H 4.42; calcd. for C_39_H_32_O_7_S_4_: C 63.22, H 4.35.

### Dipropyl-2-(2,5-bis{[4-(acetylthio)phenyl]ethynyl}-4-[(1,3-dioxo-1,3-dihydro-*2H*-indene-2-ylidene)methyl] benzylidene)-1,3-dithiole-4,5-dicarboxylate (**2**)

To a solution of **1** (100 mg, 135 μmol) and indan-1,3-dione (60.3 mg, 413 μmol) in CH_2_Cl_2_ (10 ml) was added Et_3_N (50 μl). The contents were stirred for 2 h at room temperature before the solution was filtered into stirring ether (150 ml) and was cooled for 48 h at −18 °C. The resulting precipitate was collected upon a 22 μm PTFE-filter and washed with ether, giving **2** as a dark red solid (41 mg, 35%). Melting point: 184–200 °C (decomp); TLC (CH_2_Cl_2_): *R*_f_=0.24; ^1^H NMR (500 MHz, CD_2_Cl_2_): *δ* 9.36 (s, 1H), 8.53 (s, 1H), 8.02–7.98 (m, 2H), 7.84 (dd, *J*=5.6, 3.0 Hz, 2H), 7.76–7.72 (m, 2H), 7.66–7.63 (m, 3H), 7.51–7.43 (m, 4H), 7.11 (s, 1H), 4.24–4.20 (m, 4H), 2.45 (2 × s, 6H), 1.74 (2 × sextet, *J*=7.4 Hz, 4H), 0.99 (2 × t, *J*=7.4 Hz, 6H); ^13^C APT NMR (125 MHz, CD_2_Cl_2_): *δ* 193.72, 193.60, 190.23, 189.35, 159.83, 159.66, 143.17, 142.40, 140.89, 140.81, 139.34, 137.36, 136.02, 135.92, 135.00, 134.95, 132.83, 132.74, 132.35, 131.76, 130.76, 130.41, 130.19, 129.72, 128.45, 128.16, 124.32, 123.95, 123.82, 123.68, 121.46, 111.86, 98.45, 96.46, 89.02, 88.99, 69.12, 69.04, 30.75, 30.73, 22.36, 22.34, 10.67 (1 C masked); *λ*_max_ (MeCN): 318 (*ɛ*=4.55 × 10^4^ M^−1^ cm^−1^), 361 (*ɛ*=3.35 × 10^4^ M^−1^ cm^−1^), 480 nm (*ɛ*=2.05 × 10^4^ M^−1^ cm^−1^); *λ*_max_ (CH_2_Cl_2_): 317 (*ɛ*=5.31 × 10^4^ M^−1^ cm^−1^), 363 (*ɛ*=3.91 × 10^4^ M^−1^ cm^−1^), 501 nm (*ɛ*=3.01 × 10^4^ M^−1^ cm^−1^); *λ*_max_ (PhMe): 322 (*ɛ*=3.04 × 10^4^ M^−1^ cm^−1^), 370 (*ɛ*=2.34 × 10^4^ M^−1^ cm^−1^), 504 nm (*ɛ*=1.78 × 10^4^ M^−1^ cm^−1^); HRMS (*m/z*): 869.13732 [M]^+^; calcd. for C_48_H_37_O_8_S_4_: 869.13658; Elemental analysis (%): C 66.27, 4.18; calcd. for C_48_H_36_O_8_S_4_: C 66.34, H 4.18.

## Additional information

**Accession codes:** The X-ray crystallographic coordinates for the structure reported in this Article have been deposited at the Cambridge Crystallographic Data Centre (CCDC), under deposition number CCDC 1023660. These data can be obtained free of charge from The Cambridge Crystallographic Data Centre via www.ccdc.cam.ac.uk/data_request/cif.

**How to cite this article:** Lissau, H. *et al.* Tracking molecular resonance forms of donor–acceptor push–pull molecules by single-molecule conductance experiments. *Nat. Commun.* 6:10233 doi: 10.1038/ncomms10233 (2015).

## Supplementary Material

Supplementary Information Supplementary Figures 1-10, Supplementary Table 1 and Supplementary Methods

Supplementary Data 1 X-ray crystal structure data

## Figures and Tables

**Table 1:** Best fit parameters for the Gaussian fits of the conductance histograms in Fig. 5b.

**Molecule**	***c***_***1***_**/G**_**0**_	***w***_**1**_**/log(G**_**0**_**)**	***c***_***2***_**/G**_**0**_	***w***_**2**_**/log(G**_**0**_**)**
**OPE3**	1.35 × 10^−4^	0.45±0.02		
OPE3 **1**	1.99 × 10^−4^	0.43±0.04	1.63 × 10^−5^	0.57±0.10
OPE3 **2**	1.89 × 10^−4^	0.45±0.04	2.30 × 10^−5^	0.82±0.10

**Table 2:** Calculated dipole moments (**μ**) and transmissions (*T*).

**Molecule**	Conjugation*	***μ*** **(D)**	***T***_**Fermi**_	***T***_**rel**_
**OPE3**	Linear		0.031	4.1
OPE3 **1**	Linear	5.48	0.023	3.0
OPE3 **1**	Cross	7.11		
OPE3 **2**	Linear	3.04	0.031	4.1
OPE3 **2**	Cross	5.23		
OPE3 **3**	Linear	3.61	0.035	4.6
OPE3 **4**	Inherently cross		0.0075	1
OPE3 **1**·H^+^	Cross†		0.0083	1.1

^*^The terms linear and cross do not mean that the structures correspond to the border-line resonance forms. Instead, cross means that the structure has more cross-conjugated character than does the linear structure.

^†^Cross-conjugation is here not promoted by an applied electric field, but by protonation of the aldehyde oxygen.
