# Supplementary material for: Tracking molecular resonance forms of donor–acceptor push–pull molecules by single-molecule conductance experiments
Source: Nat Commun. 2015 Dec 15;6:10233. doi: 10.1038/ncomms10233 (PMC4682163; doi:10.1038/ncomms10233)
Supplement: Supplementary Information — Supplementary Figures 1-10, Supplementary Table 1 and Supplementary Methods [file ncomms10233-s1.pdf]

Supplementary Figures

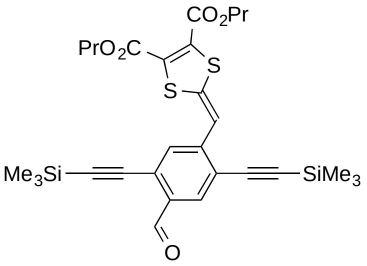

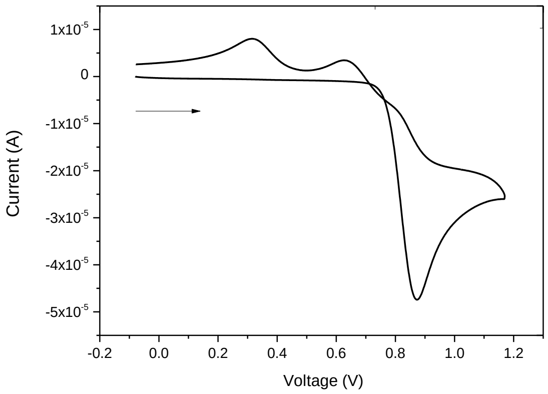

Supplementary Figure 1. Cyclic voltammogram showing irreversible oxidation of **7** (*ca.* 1 mM) at *ca.* +0.88 V *vs* Fc/Fc$^+$ in CH_2Cl_2 (+ 0.1 M Bu_4NPF_6). Conditions: Glassy-carbon disk working electrode, silver reference electrode and platinum counter electrode, scan rate 100 mV/s. The silver reference electrode was soaked in electrolyte solution. External reference: Fc/Fc$^+$ (E = 0 V) scanned at 100 mV/s.

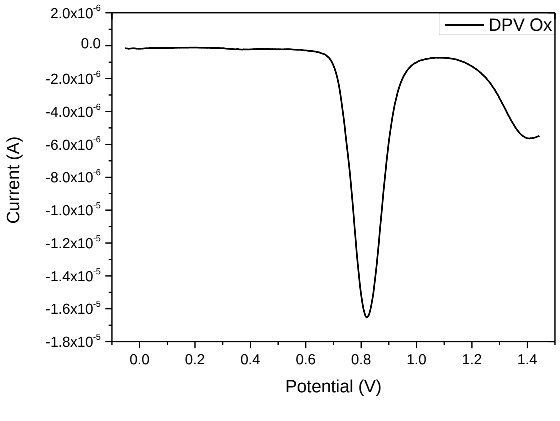

Supplementary Figure 2. Differential pulse voltammogram showing oxidation of **7** (*ca.* 1 mM) at *ca.* +0.82 V *vs* Fc/Fc$^+$ in CH_2Cl_2 (+ 0.1 M Bu_4NPF_6). Conditions: Glassy-carbon disk working electrode, silver reference electrode and platinum counter electrode. The silver reference electrode was soaked in electrolyte solution. External reference: Fc/Fc$^+$ (E = 0 V).

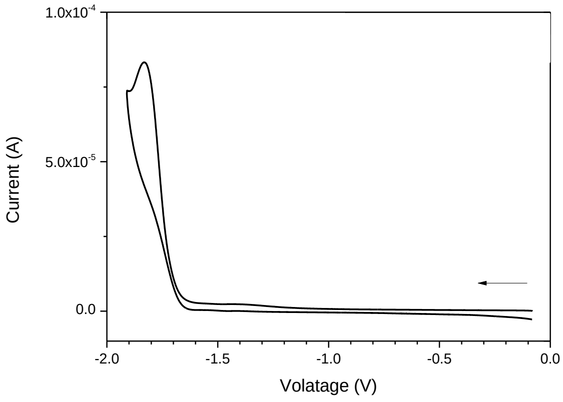

Supplementary Figure 3. Cyclic voltammogram showing irreversible reduction of **7** (*ca.* 1 mM) at *ca.* -1.84 V *vs* Fc/Fc$^+$ in CH_2Cl_2 (+ 0.1 M Bu_4NPF_6). Conditions: Glassy-carbon disk working electrode, silver reference electrode and platinum counter electrode, scan rate 100 mV/s. The silver reference electrode was soaked in electrolyte solution. External reference: Fc/Fc$^+$ (E = 0 V) scanned at 100 mV/s.

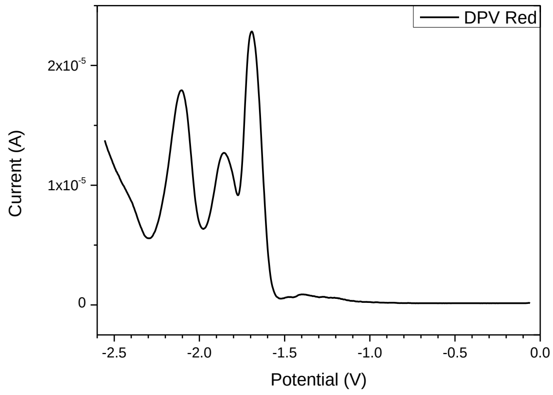

Supplementary Figure 4. Differential pulse voltammogram showing reduction of **7** (*ca.* 1 mM) at *ca.* -1.69 V *vs* Fc/Fc$^+$ in CH_2Cl_2 (+ 0.1 M Bu_4NPF_6). Conditions: Glassy-carbon disk working electrode, silver reference electrode and platinum counter electrode. The silver reference electrode was soaked in electrolyte solution. External reference: Fc/Fc$^+$ (E = 0 V).

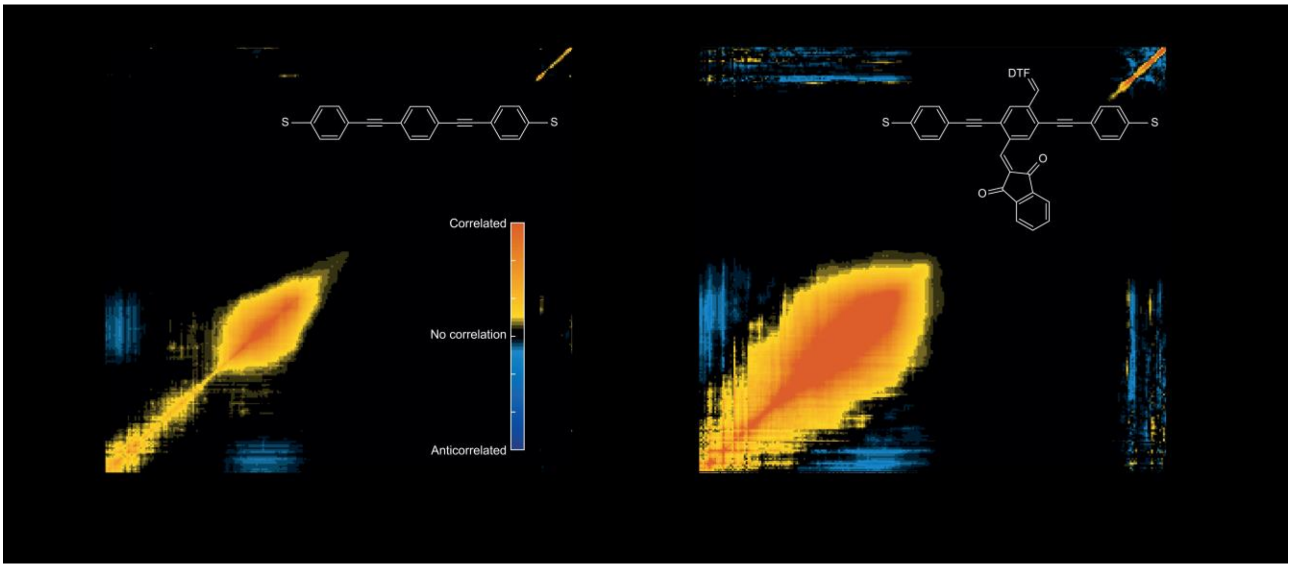

Supplementary Figure 5. Covariance matrix of **OPE3** (left) and **2** (right). Black colour indicates no correlation between conductance values, red (blue) colour represents large (anti)-correlation. We calculate the covariance matrix H_{COV} from the individual conductance breaking traces $g_i(t)$. The covariance matrix quantifies the level of correlation (or anti-correlation) between two conductance values. In each of the two plots, the region of large correlations (yellow-red) along the diagonal for conductances smaller than 10^{-3} G_0 is attributed to the plateaus of single-molecule junctions. The two anti-correlated (blue) regions at conductances of 10^{-4} G_0 - 10^{-6} G_0 indicate the steep decrease in conductance after the rupture of the molecular junctions. The correlated area around the molecular plateaus has a different size in the case of **OPE3** and **2**, related to the different behaviour of the two molecules along the plateaus. In the case of **2** the large correlation area, that goes from 10^{-3} G_0 down to 10^{-6} G_0, indicates that the molecular plateaus centred at 10^{-4} G_0 have large fluctuations (toward lower values down to 10^{-6} G_0) while in the case of **OPE3** the plateaus present much smaller fluctuations.

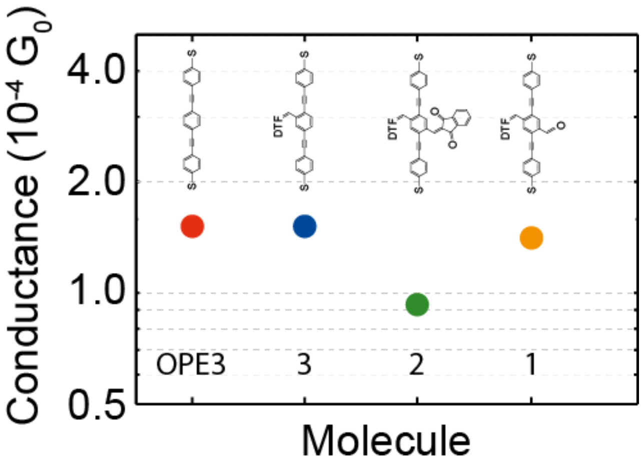

Supplementary Figure 6. Maximum of the conductance peak of the investigated molecules extracted from Gaussian fit.

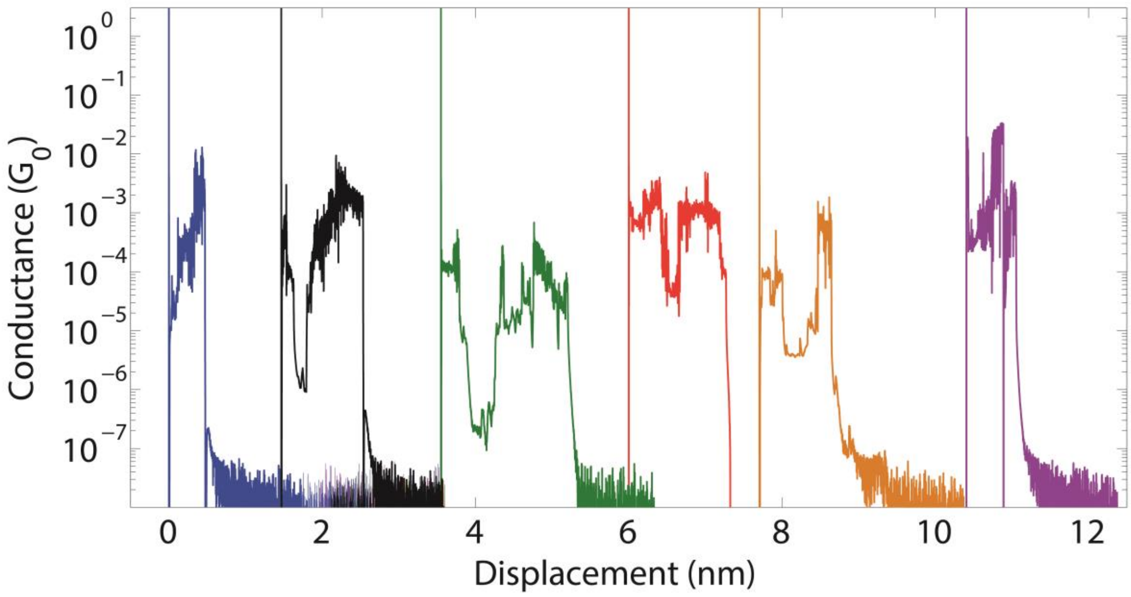

Supplementary Figure 7. Examples of individual traces for molecule **2** measured at 0.45 V of applied bias where a molecular junction is formed.

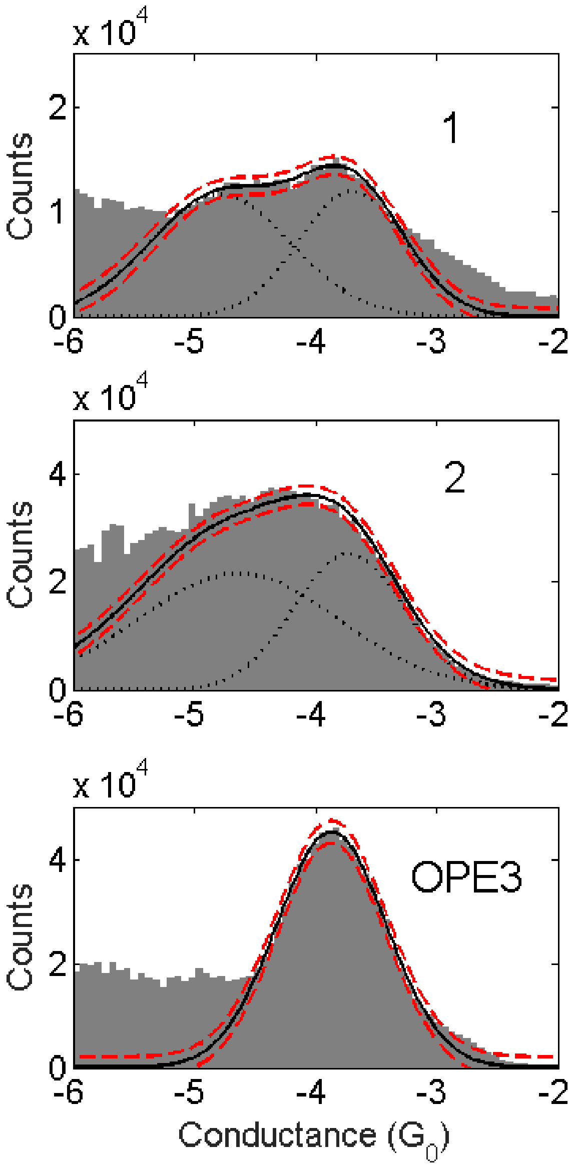

Supplementary Figure 8. Conductance histograms of **1**, **2**, and **OPE3** built from more than 1000 traces each. The thick black lines represent the fit of the molecular peak to a sum of two Gaussian curves and the dashed black lines represent the individual Gaussian peaks. The dashed red lines give 95% prediction interval of the fit.

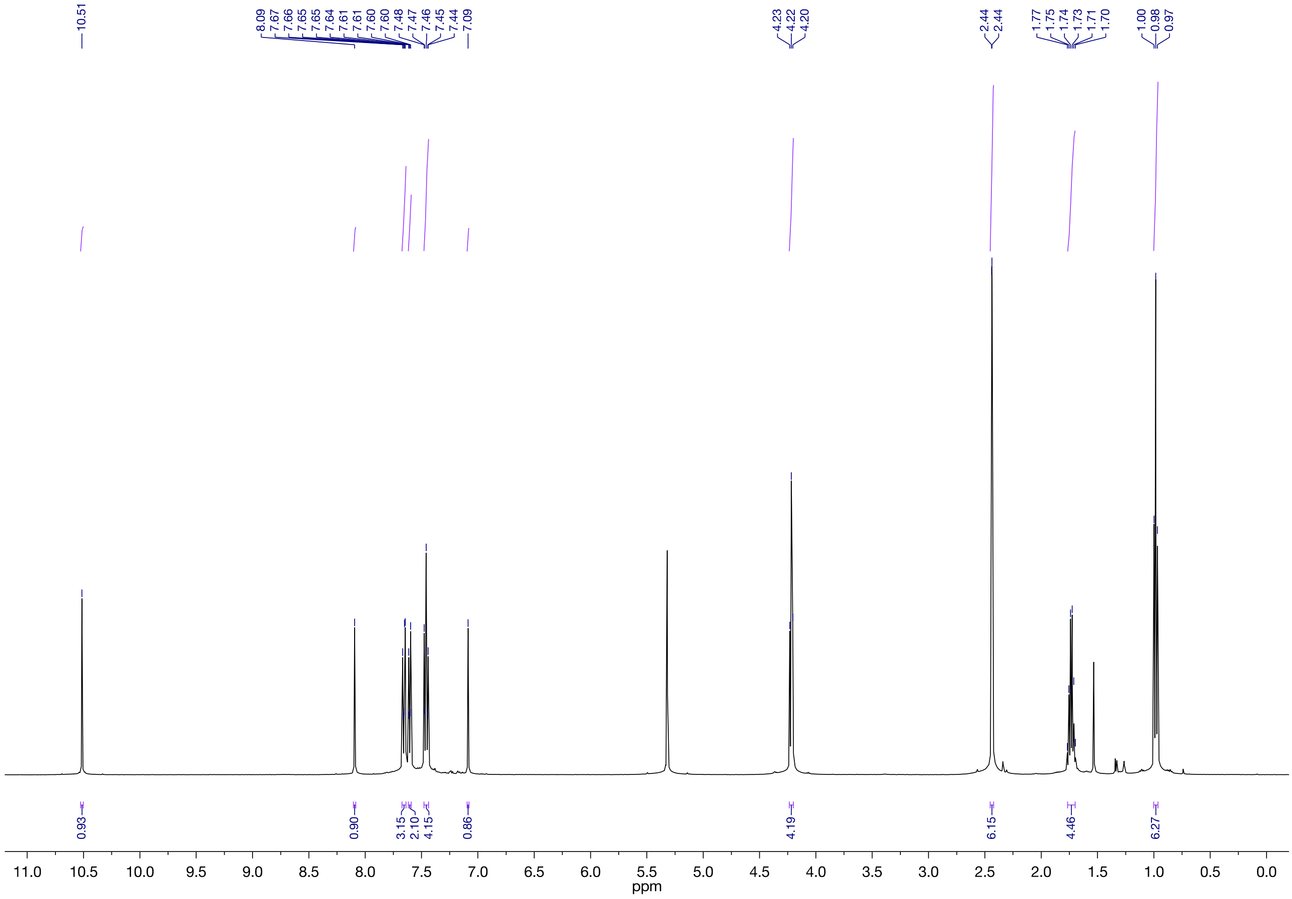

Supplementary Figure 9. ^{1}H NMR spectrum of dipropyl-2-(2,5-bis{[4-(acetylthio)phenyl]ethynyl}-4-formylbenzylidene)-1,3-dithiole-4,5-dicarboxylate **1** recorded in deuterated dichloromethane. ^{1}H NMR (500 MHz, CD_2Cl_2): δ 10.51 (s, 1H), 8.09 (s, 1H), 7.68-7.58 (m, 5H), 7.46 (m, 4H), 7.09 (s, 1H), 4.22 (t, *J* = 6.7 Hz, 4H), 2.44 (2·s, 6H), 1.73 (tq, *J* = 6.7, 7.4 Hz, 4H), 0.98 (t, *J* = 7.4 Hz, 6H) ppm.

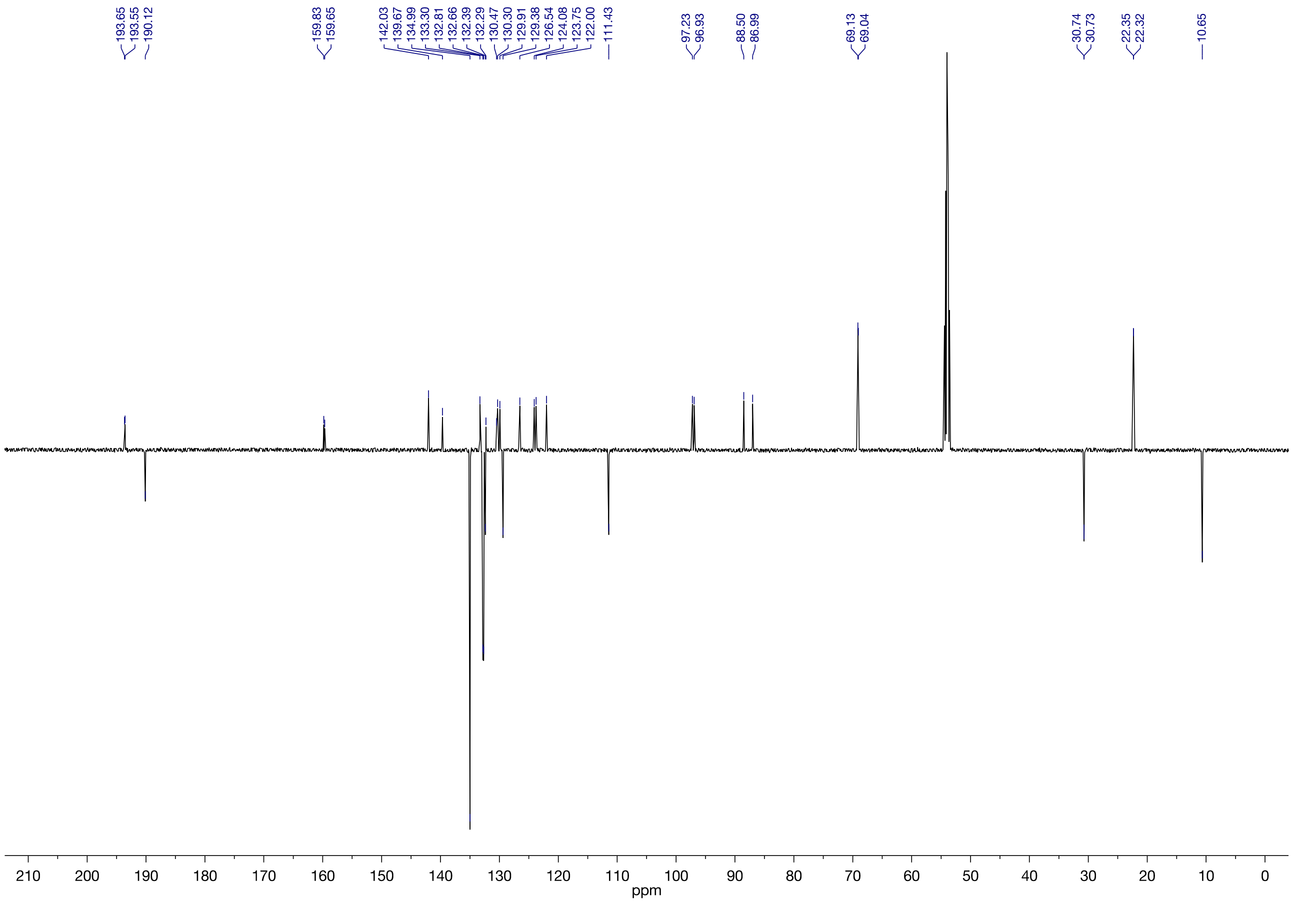

Supplementary Figure 10. ^{13}C APT NMR spectrum of dipropyl-2-(2,5-bis{[4-(acetylthio)phenyl]ethynyl}-4-formylbenzylidene)-1,3-dithiole-4,5-dicarboxylate **1** recorded in deuterated dichloromethane. CH and CH_3 correspond to negative signals and C and CH_2 correspond to positive signals. ^{13}C APT NMR (125 MHz, CD_2Cl_2): δ 193.65, 193.55, 190.12, 159.83, 159.65, 142.03, 139.67, 134.99, 133.30, 132.81, 132.66, 132.39, 132.29, 130.47, 130.30, 129.91, 129.38, 126.54, 124.08, 123.75, 122.00, 111.43, 97.23, 96.93, 88.50, 86.99, 69.13, 69.04, 30.74, 30.73, 22.35, 22.32, 10.65 ppm, (2 C masked).

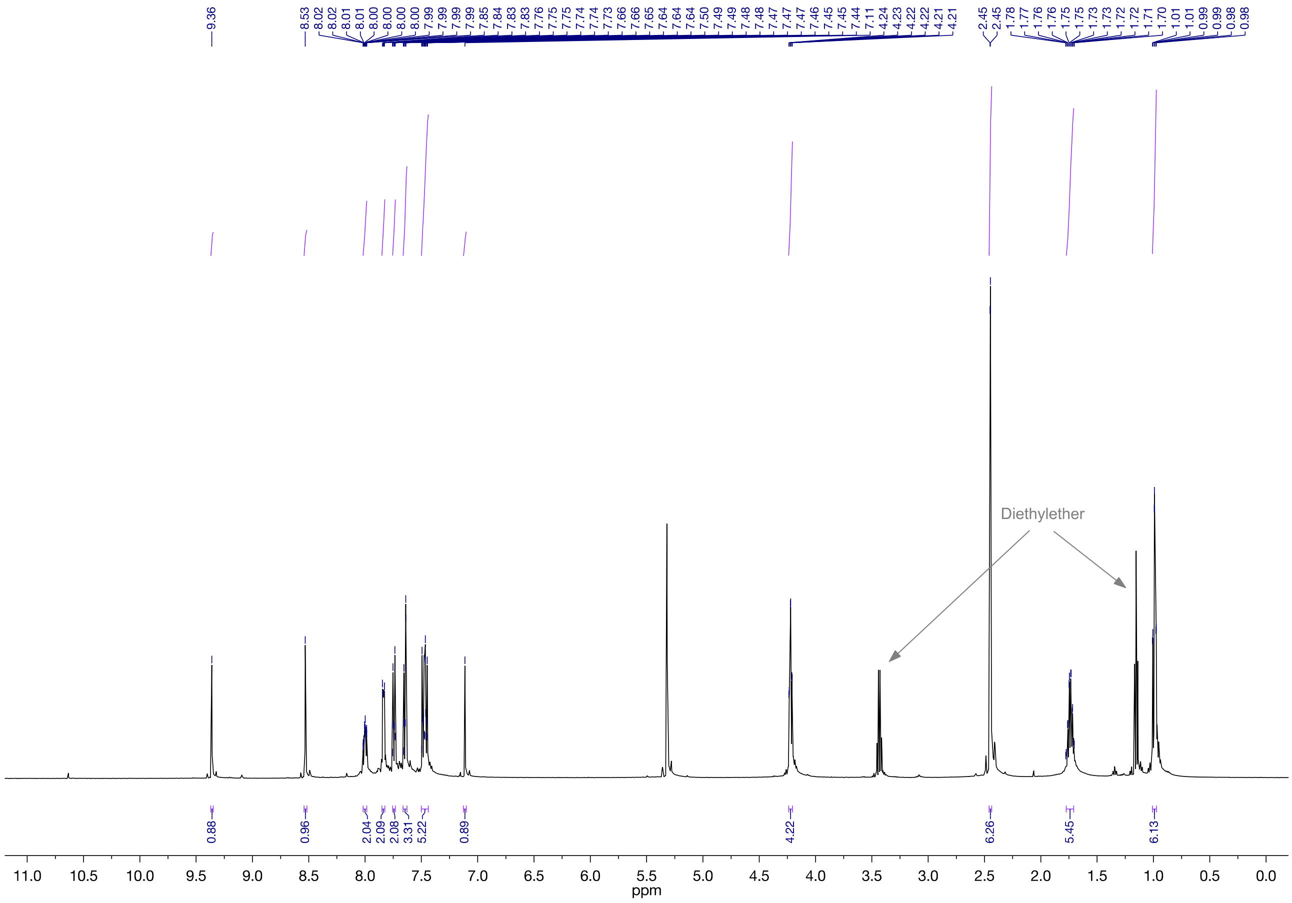

Supplementary Figure 11. ^{1}H NMR spectrum of dipropyl-2-(2,5-bis{[4-(acetylthio)phenyl]ethynyl}-4-[(1,3-doxo-1,3-dihydro-2*H*-inden-2-ylidene)methyl]benzylidene)-1,3-dithiole-4,5-dicarboxylate **2** recorded in deuterated dichloromethane. ^{1}H NMR (500 MHz, CD_2Cl_2): δ 9.36 (s, 1H), 8.53 (s, 1H), 8.02-7.98 (m, 2H), 7.84 (dd, J = 5.6, 3.0 Hz, 2H), 7.76-7.72 (m, 2H), 7.66-7.63 (m, 3H), 7.51-7.43 (m, 4H), 7.11 (s, 1H), 4.24-4.20 (m, 4H), 2.45 (2·s, 6H), 1.74 (2·sextet, J = 7.4 Hz, 4H), 0.99 (2·t, J = 7.4 Hz, 6H) ppm.

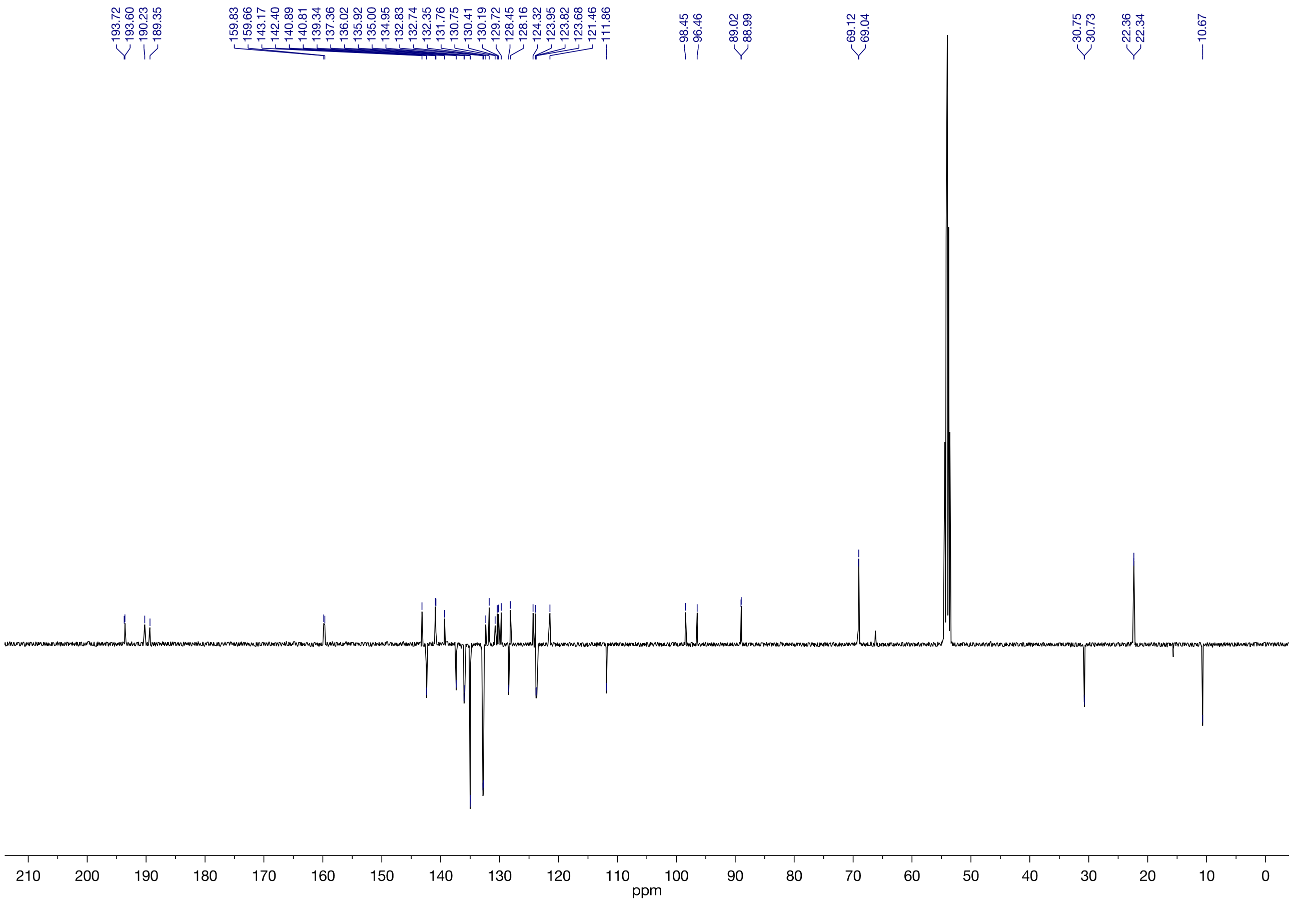

Supplementary Figure 12. ^{13}C APT NMR spectrum of dipropyl-2-(2,5-bis{[4-(acetylthio)phenyl]ethynyl}-4-[(1,3-doxo-1,3-dihydro-2*H*-inden-2-ylidene)methyl]benzylidene)-1,3-dithiole-4,5-dicarboxylate **2** recorded in deuterated dichloromethane. CH and CH_3 correspond to negative signals and C and CH_2 correspond to positive signals. ^{13}C APT NMR (125 MHz, CD_2Cl_2): δ 193.72, 193.60, 190.23, 189.35, 159.83, 159.66, 143.17, 142.40, 140.89, 140.81, 139.34, 137.36, 136.02, 135.92, 135.00, 134.95, 132.83, 132.74, 132.35, 131.76, 130.76, 130.41, 130.19, 129.72, 128.45, 128.16, 124.32, 123.95, 123.82, 123.68, 121.46, 111.86, 98.45, 96.46, 89.02, 88.99, 69.12, 69.04, 30.75, 30.73, 22.36, 22.34, 10.67 ppm, (1 C masked).

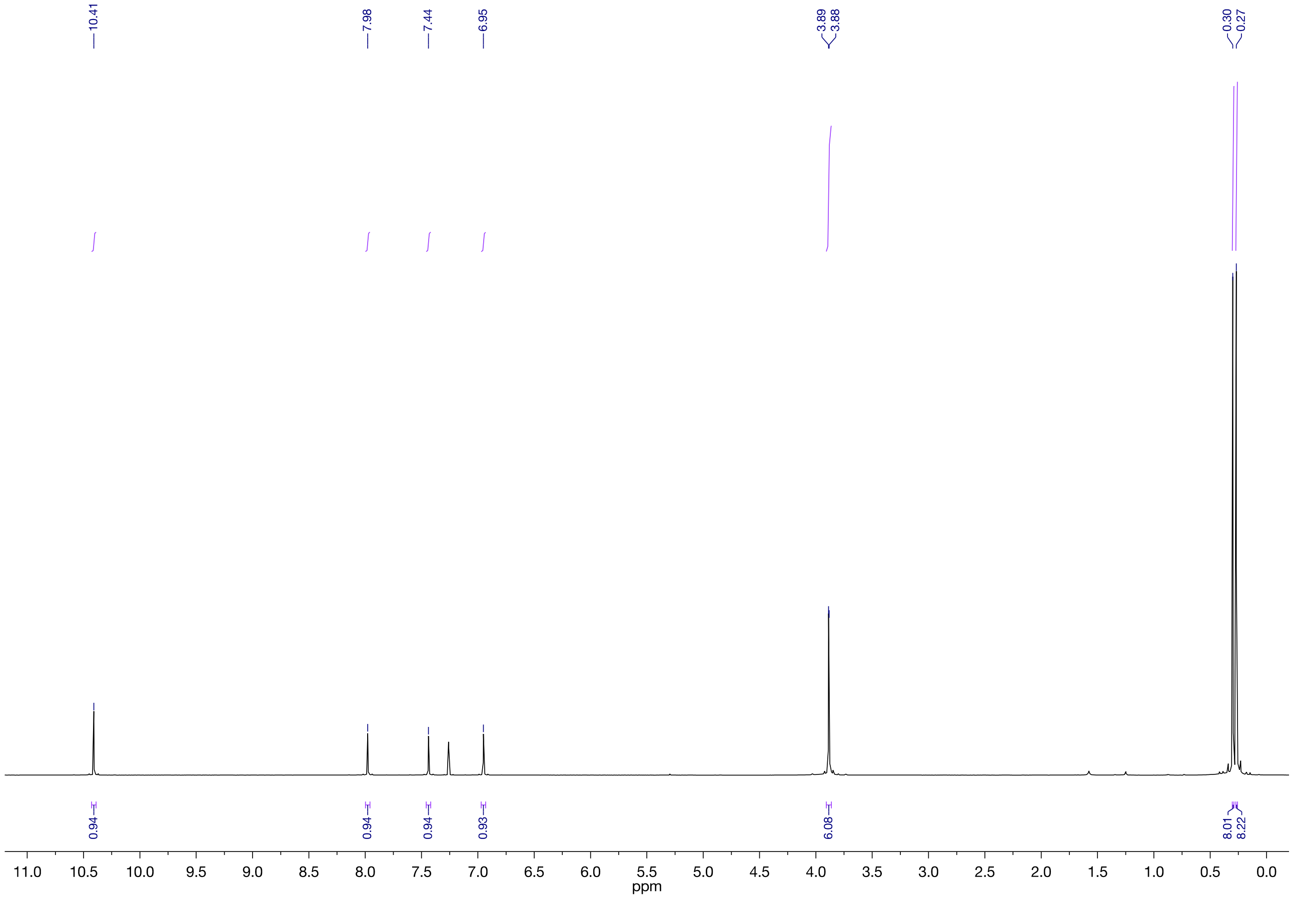

Supplementary Figure 13. ^{1}H NMR spectrum of dimethyl-2-{4-formyl-2,5-bis[(trimethylsilyl)ethynyl]benzylidene)-1,3-dithiole-4,5-dicarboxylate **7** recorded in deuterated chloroform. ^{1}H NMR (500 MHz, $CDCl_3$): δ 10.41 (s, 1H), 7.98 (s, 1H), 7.44 (s, 1H), 6.95 (s, 1H), 3.89 (s, 3H), 3.88 (s, 3H), 0.30 (s, 9H), 0.27 (s, 9H) ppm.

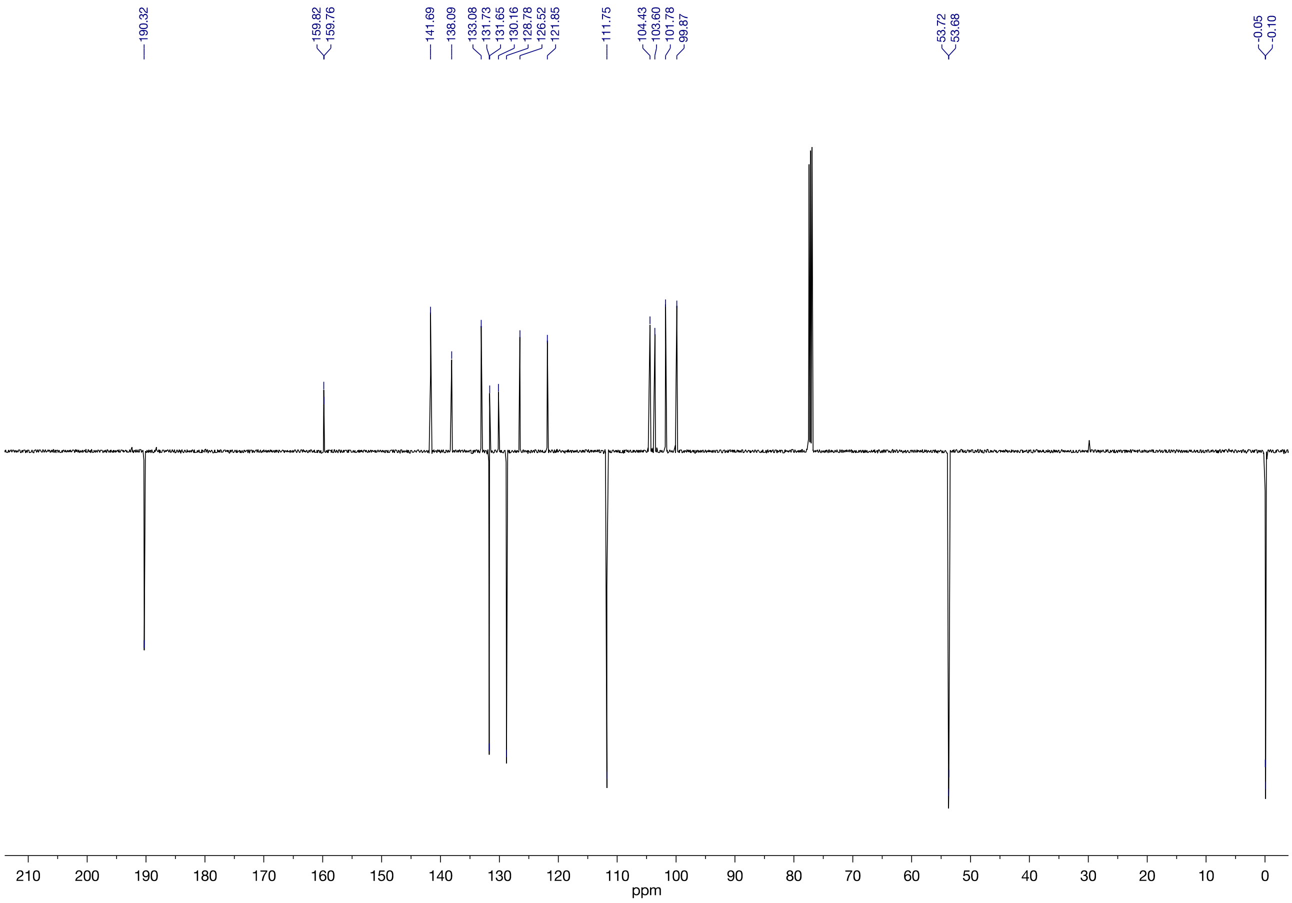

Supplementary Figure 14. ^{13}C APT NMR spectrum of dimethyl-2-{4-formyl-2,5-bis[(trimethylsilyl)ethynyl]benzylidene)-1,3-dithiole-4,5-dicarboxylate **7** recorded in deuterated chloroform. CH and CH_3 correspond to negative signals and C and CH_2 correspond to positive signals. ^{13}C APT NMR (125 MHz, $CDCl_3$): δ 190.32, 159.82, 159.76, 141.69, 138.09, 133.08, 131.73, 131.65, 130.16, 128.78, 126.52, 121.85, 111.75, 104.43, 103.60, 101.78, 99.87, 53.72, 53.68, -0.05, -0.10 ppm.

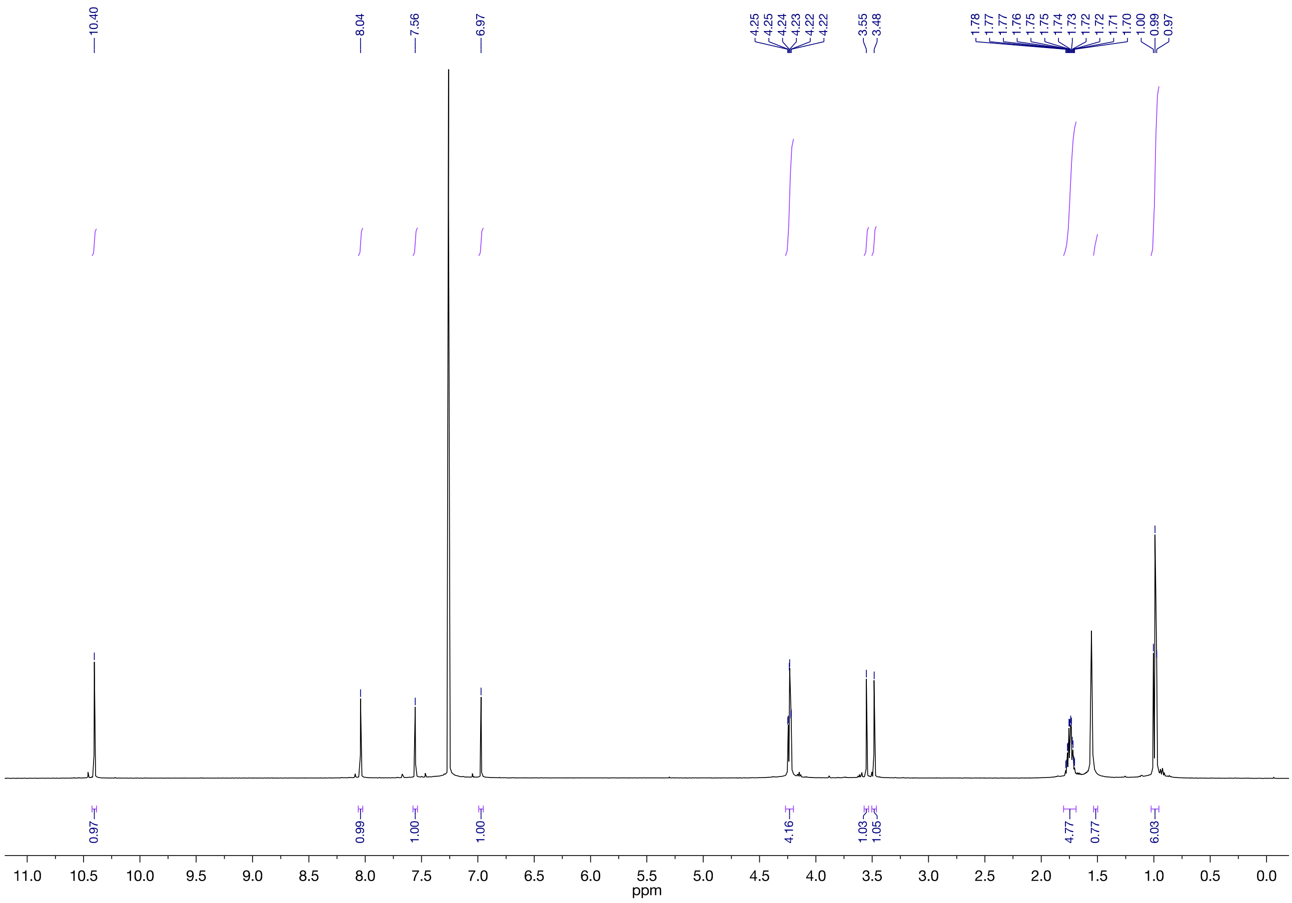

Supplementary Figure 15. ^{1}H NMR spectrum of dipropyl-2-(2,5-diethynyl-4-formylbenzylidene)-1,3-dithiole-4,5-dicarboxylate **8** recorded in deuterated chloroform. ^{1}H NMR (500 MHz, $CDCl_3$): δ 10.40 (s, 1H), 8.04 (s, 1H), 7.56 (s, 1H), 6.97 (s, 1H), 4.24 (t, J = 6.7 Hz, 2H), 4.23 (t, J = 6.7 Hz, 2H), 3.55 (s, 1H), 3.48 (s, 1H), 1.70-1.78 (m, 4H), 0.99 (t, J = 7.4 Hz, 6H) ppm.

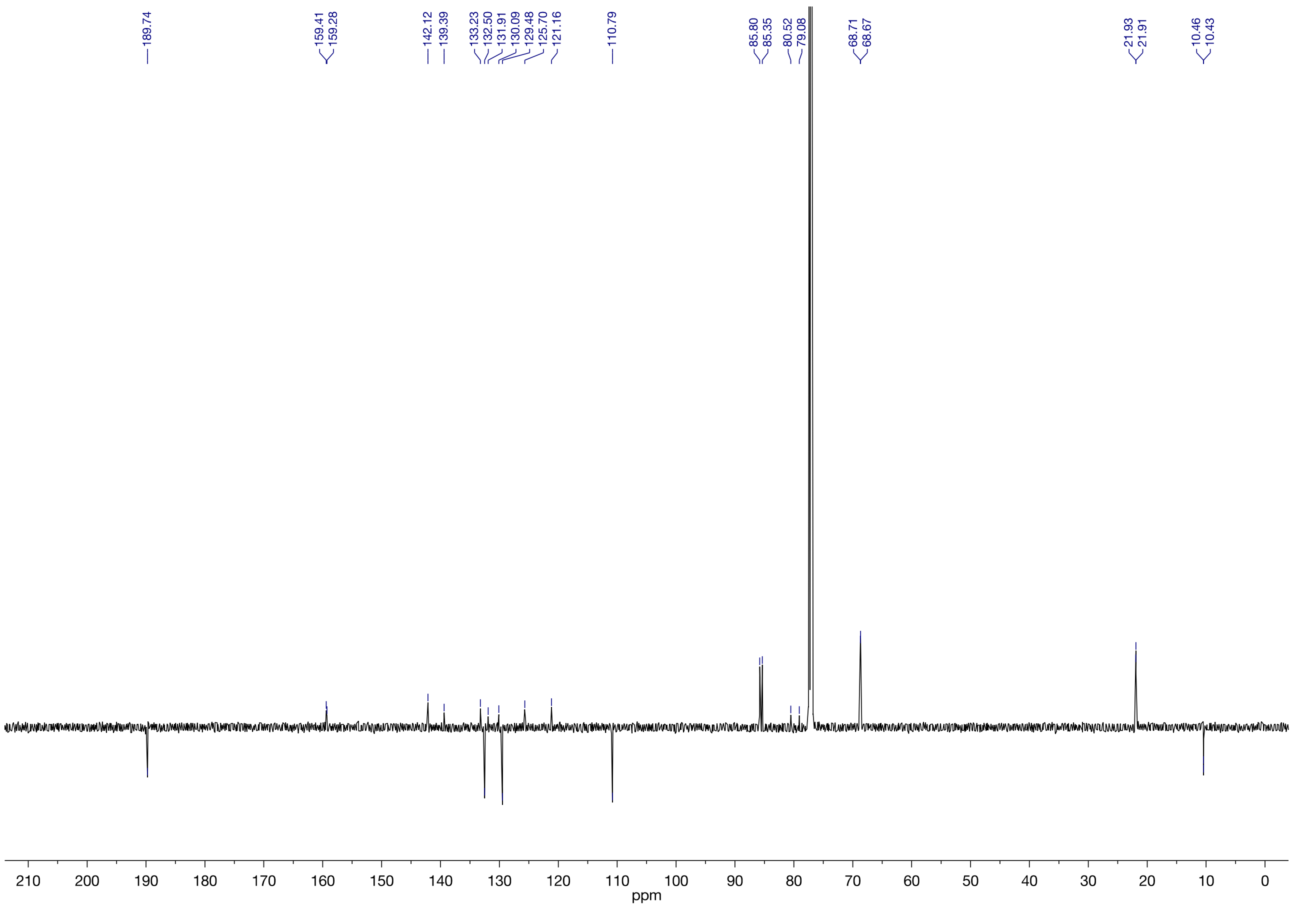

Supplementary Figure 16. ^{13}C APT NMR spectrum of dipropyl-2-(2,5-diethynyl-4-formylbenzylidene)-1,3-dithiole-4,5-dicarboxylate **8** recorded in deuterated chloroform. CH and CH_3 correspond to negative signals and C and CH_2 correspond to positive signals. ^{13}C APT NMR (125 MHz, $CDCl_3$): δ 189.74, 159.41, 159.28, 142.12, 139.39, 133.23, 132.50, 131.91, 130.09, 129.48, 125.70, 121.16, 110.79, 85.80, 85.35, 80.52, 79.08, 68.71, 68.67, 21.93, 21.91, 10.46, 10.43 ppm.

Supplementary Tables

Supplementary Table 1 | Calculated[a)] Mulliken charges for the Donor-Acceptor systems.

	Donor (S) [b)]	*Donor (S)* [b)]	*Acceptor (O)* [b)]	*Acceptor (O)* [b)]
*OPE **1** "linear"*	0.227	0.178	-0.169	
*OPE **1** "cross"*	0.241	0.185	-0.198	
*OPE **2** "linear"*	0.237	0.190	-0.185	-0.177
*OPE **2** "cross"*	0.239	0.200	-0.201	-0.200

[a)] Using DFT/CAM-B3LYP/cc-pVDZ). [b)] *Donor (S)* refers to sulfur atoms of the dithiafulvene ring, while *Acceptor (O)* refers to the oxygen(s) of the acceptor, either CHO or indan-1,3-dione.

Supplementary Methods

Fitting of Conductance Histograms: In the main text we fit the conductance histogram data to two different curves: a two-piece (asymmetric) gaussian, that has 4 fitting parameters, and the sum of two gaussians, with 6 fitting parameters in total. The fitted data are the conductance peaks from 4 conductance histograms. In the case of the two-piece gaussian, when we fit the asymmetric peaks of molecules **1**, **2** and **3**, the four fitting parameters are not redundant since the center of the distribution, the left and right width and the height of the peak are all evident from the histograms. For **OPE3**, three parameters are enough, since the peak is symmetric, and so the left and right standard deviations are the the same (as seen in Figure 5b in the main text). In the case of the sum of two gaussians, 6 parameters appear to be many but we actually impose a constraint in the fitting procedure that effectively reduces the degrees of freedom of the fit. The high conductance peak in molecules **1**, **2** and **OPE3** must have similar width and center (within 10%) and since in **OPE3** this is the only peak and is well resolved, the actual parameters of the fit pass from 6 to 4, a number not redundant anymore
